# The Need of Veterinary Education in Medical Colleges1Read before the Thirty-fourth Annual Meeting of the United States Veterinary Medical Association, Nashville, Tenn., September, 1897.

**Published:** 1897-10

**Authors:** E. P. Niles

**Affiliations:** Blacksburg, Va.


### THE NEED OF VETERINARY EDUCATION IN MEDICAL COLLEGES.[1]

BY E. P. NILES, V.S.
BLACKSBURG, VA.

WHEN the Secretary wrote asking me to present a paper at this meeting, I was at a loss for a suitable topic to write a paper on, and for one to select a subject which has not already been time-worn on all sides is no easy task. So at the very last moment, and after receiving an urgent letter from the Secretary urging me to name my subject, in order that the programme might be printed in full, I decided to occupy but a few moments of your time in presenting to you this paper. I have chosen this subject not with the hope of advancing any new ideas or recording any wonderful discoveries, but because this matter is receiving so little attention, particularly by our medical brethren.

Upon giving the subject but a passing thought, it may seem to some of you that veterinary education in medical colleges is of but little, if any, importance, and a large number of the medical frater-

[1] Read before the Thirty-fourth Annual Meeting of the United States Veterinary Medical Association, Nashville, Tenn., September, 1897.

nity may ask, What do we care for a knowledge of the diseases of the lower animals? In answer to the latter I would ask, How many lives could you have saved had you but known the source of certain contagious diseases which you have treated in your practice? The idea of employing a veterinarian as a member of the faculty of a medical college seems to have given cause for but little thought, even at the present day, and I know of but three institutions which have established a chair of comparative pathology. Until recently it could not be wondered at that more importance was not attached to veterinary education by our medical boards, for we have been laboring in our infancy—screaming and howling as only infants can—but now we are shedding our long garments and gradually reaching maturity. We are concentrating our forces. We are rapidly securing State laws in the advancement and the elevation of our profession. Our colleges are increasing their curriculums and teaching-forces. We are gaining the standing of professional men instead of the common, every-day "horse doctor." In fact, we have become educators instead of a drag upon educators. We are now capable of nursing instead of being nursed. Therefore, let us educate the public and our medical boards to the importance of this branch of science, and, if necessary, do a little nursing at the same time.

Too often a physician is called in to treat an infectious disease of the human family, and contents himself with making a simple diagnosis, and, after leaving directions as to treatment, departing without making an effort to trace the source of the disease. Can we call this negligence on his part? I hardly think so, for had he been taught comparative pathology at the college which he represents he would have known the importance of looking outside of the patient's house for the origin of the disease, and in all probability would not have left his patient until he had made a thorough investigation. The blame, then, rests upon the college and not on the physician.

There are several reasons why a veterinarian should be employed as a member of the faculty of medical colleges. A successful physician must be a good sanitarian. He must be able to prevent disease as well as to cure. He must be educated to know the value and importance of veterinary science. He must be educated to the importance of the appointment of a veterinarian on sanitary boards. He must be impressed with the fact that the two professions are closely related and, for the public welfare, must go hand in hand. He can only be so educated at the hands of the veterinarian.

A few years ago it seemed to be a matter of little importance whether a physician knew anything of the infectious diseases of the lower animals or not; but, since the researches in bacteriology have opened up a comparatively new field of science, we are forced to admit that contagious and infectious diseases are due to a specific microorganism, and that certain microörganisms are capable of causing disease in both the higher and lower animals. In many instances, however, the manifestation of disease may be so modified in the lower animals as to render it impossible for the physician who has had no training in comparative pathology to make a correct diagnosis; the physician in such cases failing to do his duty to his patients for the want of proper training. It would seem that certain branches of medical science were degenerating, although rapid strides have been made in certain directions. I refer particularly to the lack of interest shown in comparative pathology. In the beginning of the eighteenth century, when but little was known of veterinary science, Fleming states that eminent physicians devoted all their energies to the advancement of comparative pathology. These energies seem to have been peculiar to our forefathers, for where have we to-day, with the exception of three universities, a single individual, outside of the veterinary profession or an institution, manifesting any marked interest in comparative pathology? We also find that in the year 169 A.D., Galen, a noted physician of that time, is credited with having said that the education of a doctor was incomplete without a knowledge of the processes of disease among the lower animals.

Our colleges seem to have drifted into a narrow channel which, year by year, becomes deeper and narrower, and from which it is difficult to extricate them. Medical men, not having their attention called to the importance of having comparative pathology taught in their colleges, have lost sight of the early efforts of the medical profession, and are content with a knowledge of human pathology alone. They do not realize that their sanitary boards would be much more efficient and that they themselves would gain a much more prominent position in their community as public benefactors. With men who have been trained in comparative pathology as members of boards of health, diseases of the human family will be more effectually controlled. The importance of legal recognition of the veterinary profession would be more fully appreciated, and the appointment of a qualified veterinarian on all such boards would result. New positions for veterinarians would be created, and our profession would advance still more rapidly than now. Sanitary boards would become more than mere collectors of statistics. Legislators would soon see the importance of providing such boards with sufficient funds to prosecute the work of controlling and stamping out contagions and infections. In fact, these boards would become investigators and advisers. What are the majority of them now? I have in mind a city board of health which does but little beyond the employment of men to sweep the streets, and no doubt many of you could mention other similar boards of health.

The physician of this day and age should, to a certain extent, be an investigator, and in order to be an investigator in the proper sense of the term he must know comparative pathology.

Beginning with the earliest history of medicine, we find that the physician at that time gained the greater portion of his knowledge of medicines by experimentation upon the lower animals. His knowledge of anatomy of the human body was gained only by a most superficial examination of the carcass of a dead animal. He noted the arrangement of the organs and their connection with each other, and took it for granted that the organs of man must bear a close relation to those of the lower animals, and that their functions and relations to each other must be similar. He based his principles of the treatment of disease of man upon this crude knowledge of physiology and anatomy of animals. In those days experimentation upon man and the dissection of the human body were practically impossible, and it became necessary, therefore, for the physician, to a certain degree, to study veterinary science in order to obtain a practising knowledge of medicine.

At the age in which we are living, human subjects are more easily obtained; hence the anatomy of man may be studied without the aid of the lower animals, but not so with medicine. Here we still find it necessary to avail ourselves of the opportunity for study offered by animals. Actions, uses, and doses of new drugs are thus obtained. Antidotes for poisons are thus discovered, and antitoxins obtained for the treatment of disease in man. Similarity and identity of disease of man and animals are observed, and as animals furnish a very large proportion of our food we are beginning to realize more fully than ever that the source of some of the most fatal diseases of man is to be found in the lower animals.

If the two professions so closely related, yet in many respects so far divided, were marching along more nearly hand in hand, both would be bettered; both would become more successful; both would advance more rapidly; the physician would become more nearly an ideal public sanitarian, and every patient visited by him would be made to feel that he was truly in the hands of a good Samaritan. Hundreds of lives would be saved annually as a result of the practical extermination of preventable diseases, as is illustrated by the statements made by Prof. William Pepper in the *Brooklyn Medical Journal.* He says: "In Philadelphia during the past twenty years there were 55,000 deaths from tuberculosis, 11,500 from typhoid fever, 8000 from scarlet fever, and 155,000 from various infantile causes, all of which must be included in the list of preventable diseases." He classes all of these as preventable diseases, and adds: "If we should personally practice preventive medicine, I am certain our services would be vastly greater to suffering humanity than ever before. It is a higher medicine than that which seeks to quell disease after it has spread over our cities and eaten into the vitals of the people."

Here, then, we find it necessary for the physician who, for example, would prevent the spread of tuberculosis in the human family to have a sufficient knowledge of the disease in the lower animals to be able to advise his patrons in that direction; for what good is there in attempting to check the ravages of contagious diseases in man if man is to continue to be exposed to the same disease in the lower animals? Therefore, a physician can but practice preventive medicine imperfectly without first having had the proper training in both branches of the medical science. A veterinarian as a member of the faculty of every medical college is, therefore, indispensable in this day and age; and while we are making rapid strides in so many other directions, let us not lose sight of the importance of agitating this question.

